# Evaluation of daily patient positioning for radiotherapy with a commercial 3D surface-imaging system (Catalyst™)

**DOI:** 10.1186/s13014-016-0728-1

**Published:** 2016-11-24

**Authors:** F. Walter, P. Freislederer, C. Belka, C. Heinz, M. Söhn, F. Roeder

**Affiliations:** 1 Department of Radiation Oncology, University Hospital of LMU Munich, Marchioninistr 15, 81377, Munich, Germany; 2 Department of Molecular Radiation Oncology, German Cancer Research Center (DKFZ), Heidelberg, Germany

**Keywords:** Optical surface scanning, Catalyst, Patient positioning, Cone-beam computed tomography

## Abstract

**Background:**

To report our initial clinical experience with the novel surface imaging system Catalyst™ (C-RAD AB, Sweden) in connection with an Elekta Synergy linear accelerator for daily patient positioning in patients undergoing radiation therapy.

**Methods:**

We retrospectively analyzed the patient positioning of 154 fractions in 25 patients applied to thoracic, abdominal, and pelvic body regions. Patients were routinely positioned based on skin marks, shifted to the calculated isocenter position and treated after correction via cone beam CT which served as gold standard. Prior to CBCT an additional surface scan by the Catalyst™ system was performed and compared to a reference surface image cropped from the planning CT to obtain shift vectors for an optimal surface match. These shift vectors were subtracted from the vectors obtained by CBCT correction to assess the theoretical setup error that would have occurred if the patients had been positioned using solely the Catalyst™ system. The mean theoretical set up-error and its standard deviation were calculated for all measured fractions and the results were compared to patient positioning based on skin marks only.

**Results:**

Integration of the surface scan into the clinical workflow did not result in a significant time delay. Regarding the entire group, the mean setup error by using skin marks only was 0.0 ± 2.1 mm in lateral, −0.4 ± 2.4 mm in longitudinal, and 1.1 ± 2.6 mm vertical direction. The mean theoretical setup error that would have occurred using solely the Catalyst™ was −0.1 ± 2.1 mm laterally, −1.8 ± 5.4 mm longitudinally, and 1.4 ± 3.2 mm vertically. No significant difference was found in any direction. For thoracic targets the mean setup error based on the Catalyst™ was 0.6 ± 2.6 mm laterally, −5.0 ± 7.9 mm longitudinally, and 0.5 ± 3.2 mm vertically. For abdominal targets, the mean setup error was 0.3 ± 2.2 mm laterally, 2.6 ± 1.8 mm longitudinally, and 2.1 ± 5.5 mm vertically. For pelvic targets, the setup error was −0.9 ± 1.5 mm laterally, −1.7 ± 2.8 mm longitudinally, and 1.6 ± 2.2 mm vertically. A significant difference between Catalyst™ and skin mark based positioning was only observed in longitudinal direction of pelvic targets.

**Conclusion:**

Optical surface scanning using Catalyst™ seems potentially useful for daily positioning at least to complement usual imaging modalities in most patients with acceptable accuracy, although a significant improvement compared to skin mark based positioning could not be derived from the evaluated data. However, this effect seemed to be rather caused by the unexpected high accuracy of skin mark based positioning than by inaccuracy using the Catalyst™. Further on, surface registration in longitudinal axis seemed less reliable especially in pelvic localization. Therefore further prospective evaluation based on strictly predefined protocols is needed to determine the optimal scanning approaches and parameters.

## Introduction

The introduction of radiotherapy techniques with highly conformal dose distributions such as intensity modulated radiotherapy (IMRT) and stereotactic radiotherapy allows a precise application of radiation dose to the target volume with improved sparing of organs at risk. However, accurate patient positioning is crucial for the use of highly conformal radiotherapy techniques due to reduced safety margins. Therefore reliable methods of daily image guidance (IGRT) to monitor patient positioning are needed [1]. Most commonly imaging is performed using planar radiographs or cone beam CT (CBCT) which provide good information about internal anatomical structures such as bones or soft tissue. However, both techniques use ionizing radiation which should be reduced to a minimum according to the ALARA-principles [2]. Moreover these techniques lack the ability to sufficiently monitor intrafractional movements of the target caused by respiratory, cardiac or gastrointestinal motion [3].

Thus alternative imaging modalities, such as ultrasound [4, 5] or optical surface imaging [6, 7], have been successfully investigated to supplement the clinically well-established imaging modalities with special regard to intrafractional motion. Given their general advantages (fast, non-invasive and not using ionizing radiation) compared to conventional imaging, they might be beneficial also for daily patient setup.

However, the clinical applicability of optical systems may be limited by the degree of correlation between movements of the patient’s surface, the deeper located anatomical structures and the respective target volume [8]. Therefore the precondition for using an optical surface scanning system for daily setup correction would be the clinical validation of its accuracy of positioning by comparison with conventional techniques.

At our institution the novel optical surface-imaging system Catalyst™ system (C-RAD AB, Sweden) which provides applications for patient positioning, monitoring and gating, is installed on an Elekta Synergy™ (Elekta AB, Sweden). Initially, it has been introduced into our clinic mainly to establish deep-inspiration breath-hold breast cancer treatments and to investigate intrafraction motion. However, a preclinical study by Palotta et al. [9] investigated the use of a similar surface imaging system (Sentinel™, C-RAD AB, Sweden) in rigid-body phantoms also for patient positioning and found improved accuracy to detect misalignments of both optical surface imaging and CBCT compared to portal images [9]. We therefore decided to analyze the clinical data acquired during the introduction phase of the system at our institution with regard to its usability for daily patient alignment. The object of the current study was to evaluate the theoretical setup error of the 3D surface-imaging using the Catalyst™ system in combination with an Elekta Synergy™ accelerator with an Agility-MLC compared to cone beam CT based image-guidance.

## Methods

During the introduction phase of the Catalyst™ optical surface system into clinical routine, additional data on daily setup accuracy was acquired in 25 patients, which were analyzed retrospectively. This included patients with target volumes in thoracic, abdominal and pelvic body regions

### Clinical work flow

For treatment planning, patients with thoracic targets were positioned in supine position using an alpha-cradle (wingSTEP™, IT-V, Austria). Patients with abdominal targets were placed in supine position, patients with pelvic targets in either supine or prone position. For each patient a planning CT dataset was acquired using a Toshiba Aquillion LB CT Scanner (Toshiba Medical Systems Corporation, Japan) and skin marks were placed using a conventional laser-alignment system to mark to reference point. After target volume delineation and treatment planning, the structure set and treatment plan were transferred to the Catalyst™ optical surface scanner. A reference image was created for each patient using the CT-surface information and was cropped defining the region of interest on the patients’ surface individually by one treating physician.

For treatment, patients were routinely positioned on the treatment couch using the skin marks and the conventional laser-alignment system. Patients were then moved to the calculated isocenter position. With the patient in treatment position an optical scan was performed. Shift vectors for isocenter correction to achieve an optimal surface match of real time surface and the CT-based reference image were automatically calculated by the Catalyst™ software c4D (C-RAD AB, Sweden). Parameters of all three translation directions were documented and analyzed retrospectively using the analysis tool of the c4D software. CBCT was performed and the calculated translational vectors defining the setup-error based on internal anatomical structures using a clip box were documented. Patient positioning was corrected shifting the treatment couch to the optimal isocenter position based on the CBCT information and radiotherapy was performed as routinely using an Elekta Synergy™ accelerator with an Agility-MLC. All steps regarding patient treatment were done as usual according to our clinical standards. Acquisition of the treatment planning CT, target volume definition, treatment planning, dose delivery as well as type and frequency of image-guidance were not modified. Correction of patient positioning prior to treatment was solely based on CBCT.

### Optical surface scanning

**Fig. 1:**
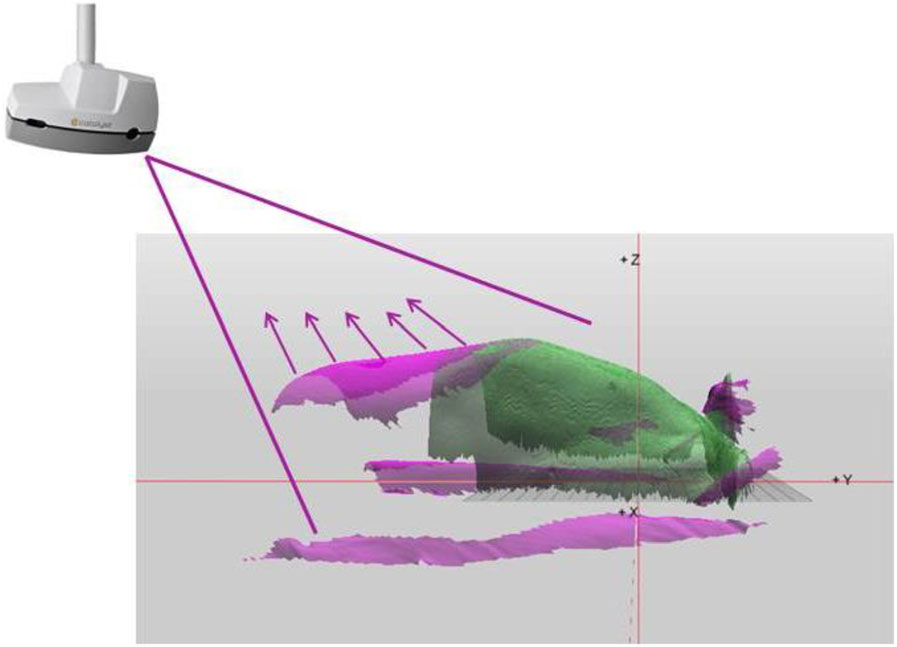
Catalyst™ setup in the treatment room

Optical surface scanning was carried out using the Catalyst™ system. A single scanning unit consisting of two components, a projector unit using light-emitting diode (LED) lights and a charge-coupled device (CCD) camera, is mounted to the ceiling in the treatment room at the foot-end of the treatment couch projecting a rapid and near-visible sequence of light patterns onto the patients’ surface (Fig. 1). Optically visible light with a wavelength of 450 (blue), 528 (green) and 624 nm (red) is used. The reflected light from the patient’s surface is captured by the camera unit. The patient’s position in the room is determined by means of optical triangulation of the reflected light and the Catalyst™ software calculates the patient’s surface using a non-rigid registration algorithm. For data acquisition we used the application “cPositioning” of the c4D software in “clinical mode”, which allows only to choose the respective patient and to adjust camera settings by a “pre-setup mode” to optimize image quality. The pre-setup mode includes predefined templates for camera settings like scan volume, tolerance values regarding the depiction of surface deviations and surface averaging time. The templates for thorax, abdomen and pelvis provided by the manufacturer were slightly adjusted for our purpose as shown in Table 1. The Catalyst™ system is a CE certified medical device and was used solely according to its objective.

**Table 1:** Camera setting Templates

Thorax	
Tolerance Settings	
lateral	5.0 mm
longitudinal	5.0 mm
vertical	5.0 mm
rotation	5.0°
roll	5.0°
pitch	5.0°
Surface Settings	
Tolerance	10 mm
Image Surface Averaging	
Time	3 s
Abdomen	
Tolerance Settings	
lateral	5.0 mm
longitudinal	5.0 mm
vertical	5.0 mm
rotation	5.0°
roll	5.0°
pitch	5.0°
Surface Settings	
Tolerance	15 mm
Image Surface Averaging	
Time	4 s
Pelvis	
Tolerance Settings	
lateral	8.0 mm
longitudinal	8.0 mm
vertical	8.0 mm
rotation	5.0°
roll	5.0°
pitch	5.0°
Surface Settings	
Tolerance	15 mm
Image Surface Averaging	
Time	3 s

Predefined templates for camera settings with tolerance values regarding the depiction of surface deviations and surface averaging time

### Statistics

The translational vectors calculated based on the CBCT (vLaser) describe the setup error made by positioning of the patient solely using skin marks and the fixed room lasers. The translational vectors calculated by the Catalyst™ software (vCatalyst) were acquired by comparison of the actual surface scan to the reference image cropped from the planning CT. Correction of the patients position according to CBCT was assumed as gold standard for patients positioning. To assess the theoretical setup error that would have occurred if the patients had been positioned using solely the Catalyst™ system (vCatalyst-only), the vectors calculated by the c4D software were subtracted from the translational vectors based on CBCT (vLaser – vCatalyst = vCatalyst-only). The mean set up-error and its standard deviation of vLaser and vCatalyst-only vectors were calculated for all measured fractions. A Wilcoxon Signed-Rank test was applied to compare vLaser and vCatalyst-only groups in the entire cohort and in subgroups according to target volume localization. *P* < 0.05 was defined as level of significance for all comparisons.

## Results

The available pre-defined templates for camera settings were reasonably useful for most patients, only single cases needed minor corrections e.g., adjustments of tolerance for vertical deviation in case of weight loss during therapy. Camera integration time and gain had to be adjusted for each patient individually depending on the patient’s skin color and therefore particular reflection properties of each patient. During routinely performed radiotherapy there was no significant delay (less than 1min) in time when performing a Catalyst™ scan. The optical scan was carried out between patient positioning and CBCT the application could be operated from a workstation at the control room and therefore did not affect the clinical patient flow.

Data of 25 patients (male 20, female five) were analyzed, mainly treated for prostate (*n* = 10), gastrointestinal (*n* = 8) and lung cancer (*n* = 4). 8 patients received radiotherapy of the thorax, four patients were treated with abdominal, and 13 with pelvic targets. All received fractionated external beam radiotherapy, and a Catalyst™ scan was performed in multiple fractions (mean 6) of each patient, resulting in 154 eligible fractions in total.

**Fig. 2:**
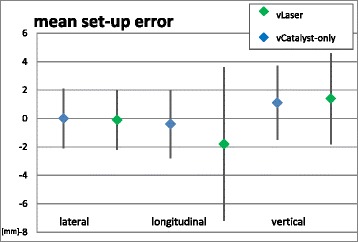
Mean setup error derived by vLaser vs. theoretical setup error by vCatalyst-only

Regarding the entire group, the mean setup error by using the laser-alignment system (vLaser) was 0.0 ± 2.1 mm in lateral, −0.4 ± 2.4 mm in longitudinal, and 1.1 ± 2.6 mm vertical direction. The mean theoretical setup error that would have occurred using solely the Catalyst™ information (vCatalyst-only) was −0.1 ± 2.1 mm laterally, −1.8 ± 5.4 mm longitudinally, and 1.4 ± 3.2 mm vertically. No significant difference was found in any direction (lateral *p* = 0.9, longitudinal *p* = 0.2, vertical *p* = 0.6). Results are visualized in Fig. 2.

Subgroup-analyses were performed for patients with thoracic, abdominal and pelvic targets (see Table 2). In patients with thoracic targets data of 25 fractions were eligible. None of the comparisons in neither axis was significant (lateral *p* = 0.6, longitudinal *p* = 0.6, vertical *p* = 0.9).

**Table 2:** Subgroup-analysis

		Thorax	Abdomen	Pelvis
Lateral	vLaser	0.7 ± 2.5 mm	2.2 ± 1.3 mm	−0.9 ± 1.4 mm
	vCatalyst-only	0.6 ± 2.6 mm	0.3 ± 2.2 mm	−0.9 ± 1.5 mm
Longitudinal	vLaser	−2.0 ± 3.5 mm	−0.4 ± 1.2 mm	0.4 ± 1.4 mm
	vCatalyst-only	−5.0 ± 7.9 mm	2.6 ± 1.8 mm	−1.7 ± 2.8 mm
Vertical	vLaser	0.6 ± 4.1 mm	2.1 ± 2.7 mm	1.0 ± 1.1 mm
	vCatalyst-only	0.5 ± 3.2 mm	2.1 ± 5.5 mm	1.6 ± 2.2 mm

Results of the subgroup-analysis for mean setup error

In 21 eligible fractions of patients with abdominal targets the mean setup error again showed no significant differences regarding the lateral (*p* = 0.5), longitudinal (*p* = 0.07) or vertical (*p* = 1) axis, although a trend was present in longitudinal direction.

**Fig. 3:**
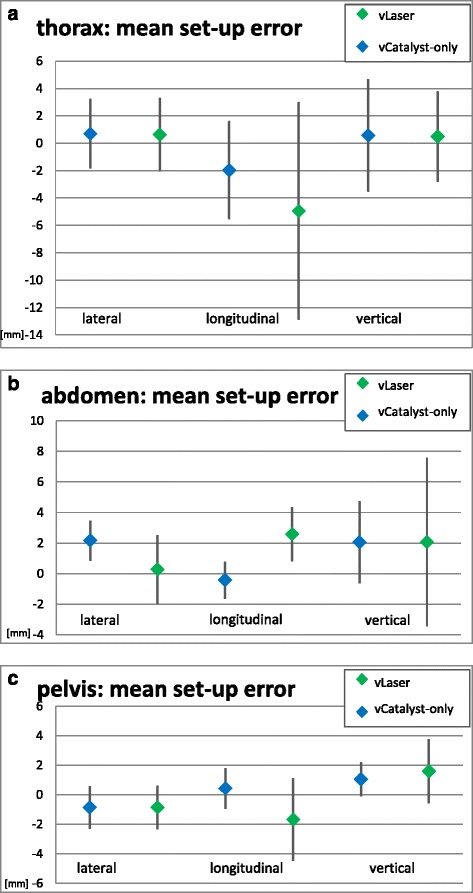
**a** Subgrup: thorax, setup error derived by vLaser vs. theoretical setup error by vCatalyst-only. **b** Subgroup: abdomen, setup error derived by vLaser vs. theoretical setup error by vCatalyst-only. **c** Subgroup: pelvis, setup error derived by vLaser vs. theoretical setup error by vCatalyst-only

In patients with pelvic targets, a total of 108 fractions were eligible. In contrast to the other body regions, we observed a significant difference favouring vLaser in longitudinal direction (*p* = 0.02), while differences were not significant in lateral (*p* = 0.9) or vertical (*p* = 0.4) axis. Results are visualized in Fig. 3.

## Discussion

Our institution is one of the first to routinely use an installation of the novel surface imaging system Catalyst™ in connection with an Elekta Synergy™ linear accelerator via the Elekta Response™ Interface. The system was implemented at our site mainly for breath-hold radiation techniques in breast cancer patients after our initial study focusing on technical characteristics such as dose delivery accuracy and time delay showed that respiratory motion is adequately assessed [7]. In contrast, the current work focuses on its usability for daily patient positioning and shows good accordance between the theoretical setup error made by the optical scanning system and the setup error made by positioning based on skin marks in general, although some differences in subgroups of patients depending mainly on tumor localization became evident.

Optical scanning systems have shown generally good agreement with conventional imaging modalities in patient positioning according to a number of studies by other groups [6, 8–12], although clinical data on the Catalyst™ system itself is still rare. Previously to the Catalyst™, C-RAD AB introduced the Sentinel™ system which has been evaluated in several studies. While the Catalyst™ uses optical light to image the entire surface of its target, the Sentinel™ uses a laser scanner to sample the surface line-by-line. By observing the whole surface via one camera, the Catalyst™ is able to calculate the surface in real time, whereas the Sentinel™ reconstructs the patients’ surface from single line projections at different time points, resulting in larger latency.

One study evaluating the Sentinel™ in a rigid body phantom showed a precision as high as 1 mm in all three axes and 1° rotation [9] if a Sentinel™ image was used as reference. The results were comparable to those obtained by CBCT and seemed to be improved compared to conventional portal imaging systems. However, when an external surface extracted from a CT was used as reference, global worsening of Sentinel™ performance occurred. A clinical study by Stieler et al. investigated the accuracy of the Sentinel™ scanner in patients with targets in different treatment regions [10]. They performed the optical scan after the correction of the patient position according to the CBCT information. Ideally, the shift vector found by the Sentinel™ should then be zero because the surface matching yields the same results provided by the CBCT. In total, the recorded disagreement in 153 analyzed fractions was −1.0 ± 3.6 mm in lateral, 1.0 ± 6.3 mm in longitudinal and −1.8 ± 5.9 mm in vertical direction in their study, with pelvic targets showing the worst disagreement compared to thoracic and head and neck cases. Thus, the authors concluded a generally good agreement between the Sentinel™ and CBCT [10]. In a subsequent study, the same group evaluated the Catalyst™ system in a similar manner [11]. After quantifying positioning accuracy and reproducibility in phantom tests, they analyzed 224 fractions in patients with head and neck, thoracic and pelvic targets. The recorded overall disagreement was 0.7 ± 2.8 mm in lateral, −1.3 ± 4 mm in longitudinal and 1.5 ± 3.6 mm in vertical direction, which seemed similar to the Sentinel™ system regarding mean values but slightly improved regarding standard deviations indicating a smaller statistical error [11].

Other optical surface scanning systems like AlignRT™ (VisionRT, London, UK) or Time-of flight cameras have also been evaluated [13–15]. For example Krengli et al. [13] reported a mean random setup error of 1.2 ± 2.3 mm along the X axis, 0.0 ± 1.4 mm along the Y axis, and 2.0 ± 1.8 mm along the Z axis and mean systematic errors of and 0.3 ± 3.0 mm, 0.5 ± 2.0 mm, and −0.7 ± 2.4 mm respectively compared to EPID in 16 patient with prostate cancer. De Antonio et al. [14] evaluated the same system for positioning in15 breast cancer patients. Systematic error was reported for vertical axis 0.12 ± 0.26 mm, longitudinal 0.07 ± 0.17, random error was 0.16 ± 0.06 and 0.18 ± 0.07 mm respectively (Deantonio et al. 2011 [14]). Unfortunately, both groups did not clearly state how they defined “systematic” and “random” error and did not state values for total error, thus making a direct comparison difficult.

In our study analyzing 154 fractions using the Catalyst™ system, we observed a smaller absolute disagreement in lateral (−0.1 ± 2.1 mm) but similar values in longitudinal (−1.8 ± 5.4 mm) and vertical (1.4 ± 3.2 mm) direction compared to the reports by Stieler et al. [10, 11]. Both studies identified the largest shifts in longitudinal and vertical direction, probably due to respiratory movement. In their subgroup analyses, Stieler et al. [10, 11] observed the largest absolute differences in pelvic targets and argued that most patients use predominantly abdominal respiration when positioned in supine causing those large deviations. Similarly, Pallota et al. [9] found larger deviations in pelvic (lateral 0.1 ± 2.5 mm; longitudinal −1.4 ± 4.0 mm; vertical −1.6 ± 3.1 mm) than in thoracic targets (lateral −0.3 ± 2.7 mm; longitudinal 0.0 ± 3.8 mm; vertical 1.3 ± 2.7 mm) comparing patient positioning by the Sentinel™ system to CBCT or portal images. In contrast, the largest absolute differences in our study were evident in thoracic targets. Similarly, Wikstrom et al. [8] could not confirm large deviations focusing on pelvic targets (lateral 0.1 ± 1,7 mm; longitudinal 0.0 ± 2.1 mm; vertical 0.1 ± 1.7 mm) in their study comparing patient positioning with the Sentinel™ to CBCT. Thus it might be more reasonable to suppose that respiratory movements are most pronounced in the thoracic region, although it cannot be ruled out that differences in the scanning method or the used reference method (entire surface vs line by line) influenced the results. Regarding the reference method, our group, similarly to Pallotta et al. [9], used the outline of the planning CT as reference for the optical scan. In contrast, Wikstrom et al. [8] used an initial surface scan by the Sentinel™ system after skin mark based positioning of the patient and already performed CBCT correction prior to the first fraction as reference for further measurements and compared this method to the use of a planning CT reference. The theoretical benefit of using a CT reference is the linkage of the CT to the treatment plan which allows not only a verification of pose and position of the patient by surface imaging but also an indirect verification of the skin marks in relation to the isocenter prior to radiation delivery [8]. However, using the initial surface imaging as reference, they observed less deviations to the CBCT registrations compared to the deviations between surface registrations based on the planning CT reference and CBCT registrations [8]. Moser et al. [12] also showed that delineation of the outline from the treatment planning system may not correspond to the surface detected by an optical scanning system. In their study, they presented large deviations between surface registrations and megavoltage CBCT registrations using the planning CT reference method. Thus it seems possible that some of the observed differences regarding the distinct target areas rather depend on the used methodology than representing true influencing factors for the applicability of surface scanning for position per se. Similarly we observed the only statistically significant difference in patients with pelvic targets. However, the largest absolute differences in our study were evident in thoracic targets (although not statistically significant probably due to the far lower number of analyzed fractions compared to pelvic targets).

We also analyzed the accuracy of the patient positioning using surface scanning with the Catalyst™ compared to positioning solely by skin marks. Regarding all patients, the mean theoretical setup error made by the Catalyst™ system did not differ significantly from the setup error made by positioning the patient on skin marks alone, although the standard deviation of the set up error in especially in longitudinal direction seemed comparably larger with Catalyst™. However, according to the subgroup analyses by target area, we observed a significant difference between Catalyst™ based set up and skin mark based set up in longitudinal direction in pelvic targets favoring skin marks. Palotta et al. [9] described a similar finding in their study but used a different method for comparison. For each patient, they recorded for the first four fractions if a setup correction with surface scanning or portal imaging resulted in improvement or worsening of the position based on skin marks only using correction with CBCT as gold standard. In patients with thoracic targets, the use of surface imaging resulted in improved positioning in 50% and in worsened positioning in 16% compared to the use of skin marks only. Use of portal images yielded similar results. However, in patients with pelvic targets surface imaging resulted in improvements in 45% but in worsening in 23%, while portal imaging yielded clearly superior results. Poorer results were particularly seen along the longitudinal and vertical axis using surface scanning in both areas. The authors speculated that the more symmetrical shape of the pelvis, the presence of hair which reduces the quality of the acquired surface image, and affections of the external body surface by different levels of bladder and bowel filling may explain the worse registration results based on surface scanning in the pelvic region.

Of course our study has some limitations: Because the data was recorded during the introduction phase of the Catalyst™ system into clinical routine for motion management, data acquisition regarding positioning did not follow a strictly predefined protocol. Therefore data had to be analyzed retrospectively and the number of imaged fractions per target area was clearly unbalanced.

Nevertheless, our study is one of the first reporting clinical data on the accuracy of patient positioning using the Catalyst™ system, which in contrast to former surface scanning systems detects the entire surface at once compared to a line by line scanning as done for example by the Sentinel™ system.

## Conclusion

In summary, our data suggest that optical surface scanning by the Catalyst™ can be used for daily positioning at least to complement complementing conventional imaging modalities. The accuracy of the system seems acceptable compared to patient positioning based on skin marks only. However, given the unexpected high accuracy of shin mark-based positioning [16] no significant improvement of patient positioning by using the Catalyst™ could be derived from the evaluated data. This underlines the high quality of patient positioning which can be achieved with skin mark based positioning if extensively used and well-trained. Further on we observed that registration in longitudinal axis is less reliable especially in pelvic localization. Therefore further prospective evaluation based on strictly predefined protocols is needed to determine the optimal scanning approaches and parameters, for example with regard to the use of an optical reference image instead of a surface image reconstructed from the planning CT as advocated by others [8]. This could also clarify the inconsistent findings regarding patient subgroups (for example with pelvic targets) which seem less suitable for positioning by surface scanning per se.
